# Evaluation of the use of decision-support software in carcino-embryonic antigen (CEA)-based follow-up of patients with colorectal cancer

**DOI:** 10.1186/1472-6947-12-14

**Published:** 2012-03-05

**Authors:** Charlotte J Verberne, Cornelis H Nijboer, Geertruida H de Bock, Irene Grossmann, Theo Wiggers, Klaas Havenga

**Affiliations:** 1 Department of Surgery, University Medical Center Groningen, PO Box 30001, 9700 RB Groningen, the Netherlands; 2 Department of Epidemiology, University Medical Center Groningen, PO Box 30001, 9700 RB Groningen, the Netherlands; 3 Department of Surgery, Catharina Hospital, PO Box 1350, 5602 ZA Eindhoven, the Netherlands

**Keywords:** follow-up, colorectal cancer, workflow automation

## Abstract

**Background:**

The present paper is a first evaluation of the use of "CEAwatch", a clinical support software system for surgeons for the follow-up of colorectal cancer (CRC) patients. This system gathers Carcino-Embryonic Antigen (CEA) values and automatically returns a recommendation based on the latest values.

**Methods:**

Consecutive patients receiving follow-up care for CRC fulfilling our in- and exclusion criteria were identified to participate in this study. From August 2008, when the software was introduced, patients were asked to undergo the software-supported follow-up. Safety of the follow-up, experiences of working with the software, and technical issues were analyzed.

**Results:**

245 patients were identified. The software-supported group contained 184 patients; the control group contained 61 patients. The software was safe in finding the same amount of recurrent disease with fewer outpatient visits, and revealed few technical problems. Clinicians experienced a decrease in follow-up workload of up to 50% with high adherence to the follow-up scheme.

**Conclusion:**

CEAwatch is an efficient software tool helping clinicians working with large numbers of follow-up patients. The number of outpatient visits can safely be reduced, thus significantly decreasing workload for clinicians.

## Background

Approximately two-thirds of patients with colorectal cancer (CRC) present with potentially curable non-metastasized disease. After completion of treatment, most CRC patients are followed up to detect recurrence, either local or distant, at an early, curable stage [1]. In our hospital, with about 100 new cases of curable CRC a year and 5 years of follow-up with outpatient appointments, the surveillance of such a cohort can accumulate to about 1200 visits, costing a great deal of time and money.

The ideal follow-up for CRC patients has not yet been settled, although there are national guidelines (http://www.oncoline.nl) [2]. Since the value of physical examination in finding recurrent disease is low, there can be a benefit in reducing outpatient visits. Current follow-up schemes focus on the most effective combination of imaging modalities and laboratory measurements. A new strategy is a low-cost 'triage' blood biomarker Carcino-Embryonic Antigen (CEA), which triggers and directs selective imaging. The keys to the effective use of CEA are frequent assessment and an interpretation based on both the absolute value and its rise [3,4]. The average normal value of serum-CEA is 2.0-2.5 ng/L. The inter-individual variation is approximately 55% [5], so that every measurement must be referenced to the previous value.

In our hospital we adhere to a CRC follow-up protocol based on CEA testing and outpatient visits every 3 months for the first three years, and every 6 months for the following two years. If the CEA value is found to have increased (>20%), the patient is requested to come to the laboratory again in 6 weeks. If a large rise (>40%) is present, or in case of a significant increase two times in a row, the patient is called up to plan Computed Tomography (CT) scanning of thorax and abdomen. Follow-up visits can renew the patients' worry about the eventual course of the disease and the outpatient clinic appointments put a strain on hospital logistics. Since CEA measurements can be taken in local laboratories without combining this with an outpatient visit to the hospital, our follow-up with selective, triggered imaging can possibly be managed using software support. For that purpose, we constructed and implemented a software system: "CEAwatch". The aim of this article was to evaluate the use and clinical value of "CEAwatch" as workflow automation.

## Implementation

### Patients

All patients curatively treated for CRC in our hospital between January 2004 and January 2010, and receiving follow-up at the time of the introduction of the software program, were extracted from the cancer registry database. This search resulted in 331 patients. Eleven patients (3%) were lost to follow-up, 63 patients (19%) already had metastatic disease at the introduction of the software, and 12 patients (4%) were not yet in follow-up after their resection. As a result, a cohort of 245 patients was constructed. Data on follow-up were obtained from the medical records.

### Software description

CEAwatch is an intranet-based software system written to support clinicians working with large numbers of patients in follow-up. The program was made using open source internet software: MySQL database, PHP scripting language, and JAVA programming language. It is deployed on a server behind the hospital's firewall. Entrance to the program is protected with a username and password. A full description of the software is added as Additional file 1.

Every day, the program extracts the latest CEA values of a given list of patients from the hospital information system and calculates the increase on the last value per patient. CEAwatch generates letters to patients giving information about the latest CEA value and its implications. If the CEA value is steady, the patient is invited to come to the laboratory again in three or six months; if the CEA value has increased (>20%), the patient is requested to come to the laboratory again in 6 weeks. In case of a significant increase two times in a row or a large increase after 3 months (>40%), the patient is called up to plan a CT scan. CEAwatch also suggests a local laboratory where the patient can have blood drawn. The clinicians double-check that the letters generated by CEAwatch fit the patient's pattern in CEA and approve the generated letters, which are then printed and mailed by the secretary of the surgical outpatient ward.

### Follow-up schedule

From 1-8-2008, when the software was introduced, patients receiving follow-up were asked to participate in the follow-up using our software system CEAwatch during their regular outpatient clinic visit. Patients who were willing to participate received patient information before beginning. We refer to this group as the study group. Those who were not asked or were not willing to participate served as the reference group. There was no randomisation between groups, because both groups received the same follow-up in terms of diagnostics and imaging, the same follow-up by other specialists (i.e., oncologists) if applicable, and clinical management in case of suspicion of recurrent disease was the same in the study and reference groups. The only difference was the use of software between groups; which resulted in the situation that patients in the study group were seen at the outpatient clinic once annually instead of four times a year and they had the possibility of having blood drawn at local laboratories instead of in the hospital. These differences were clearly explained to all patients. Colonoscopy was performed 3 years after the resection of the primay tumor according to the Dutch guideline in all patients. In each patient, an annual CT scan of thorax and abdomen was made. In case of blood sampling at local laboratories, the sample was sent to our clinic to avoid inter-assay differences. For this goal, we contacted the external laboratories in our region to send CEA samples from study patients to our laboratorium. New eligible patients were added to the database every week. When the software had been in use for 1.5 years, all data on both groups were analyzed.

### Statistical analysis

The primary goal of this study was to assess the functionality of CEAwatch and the experiences with CEAwatch of doctors. Interviews were held with the clinicians; one person (CV) interviewed nine surgeons using the software, in a face-to-face meeting. The focus of this interview was on the decrease of workload as a result of having fewer patients in follow-up at outpatient clinics. Decrease of workload was expressed in a visual analogue scale and the interview was constructed by the authors, including an epidemiologist, using face validity.

Secondary, we were interested in outcomes in terms of safety in finding metastases and local recurrences. If suspicion on metastases and recurrences rose, ascertainment or evaluations by other clinicians beyond the surgeons were assessed in both groups if applicable. Differences between both groups and follow-up data were calculated using the Chi square test and the Man Whitney *U *test.

## Results and discussion

### Patients

Of the 245 patients in the cohort, 185 patients were asked between 1-8-2008 and 1-4-2010 by their surgeon to enter CEAwatch; 184 (75.1%) patients consented. Sixty-one patients (24.9%) underwent follow-up without the software support. In Table 1 the CEAwatch group is compared with the control group regarding baseline characteristics. There were no differences between age, sex, or tumour stage between groups. Patients in the CEAwatch group had less major comorbidity and had more often rectal cancer.

**Table 1:** Patient and tumour characteristics

		CEAwatch	Usual care	P
Total (N)		184	61	NA
Male (% )		109 (59)	28 (60)	ns
Median age (range)		67.0 (38-87)	70.5 (50-88)	ns
Comorbidity (%)	Major	20 (11)	14 (25)	0.006
	Minor/None	164 (89)	46 (75)	
pT (%)	0, 1, or 2	62 (33)	13 (21)	ns
	3 or 4	121 (66)	47 (77)	
	unknown	1 (1)	1 (2)	
pN (%)	0	119 (65)	32 (52)	ns
	1 or 2	65 (35)	29 (48)	
Location (%)	Colon	98 (53)	44 (72)	0.009
	Rectum	86 (47)	17 (28)	

NA = not applicable, ns = not significant

### Efficacy of the software

The software showed few technical problems, which could all be solved instantly. The amount of time spent on follow-up patients in regular office hours decreased sharply, with percentages of up to 50% reported in the interviews with surgeons. The automatically generated letters to patients were considered an important relief and to reduce administration time. As expected, the number of outpatient clinic visits was significantly lower in the CEAwatch group. Results for follow-up are shown in Table 2.

**Table 2:** Results of follow-up and clinical visits 1 = median follow-up time in years, starting from the introduction of the new follow-up scheme; 2 = number of outpatient clinical visits to the surgeon for follow-up of CRC

	CEAwatch	Usual Care	P
Median study follow-up time^1 ^(range)	0.96 ( 0.10-1.39 )	1.34 (0.10-1.66)	0.01
Median total follow-up time (range)	2.66 (0.2-10.8 )	3.21 (0.10-6.05)	0.35
Outpatient clinics^2^	0 ( 0-7 )	3 ( 0-10 )	<0.001
Metastases found in follow-up (%)	16 (9)	8 (13)	0.06
Curative metastasectomy	7/16	1/8	0.13

In our control group, an outpatient follow-up control visit of 15 min is planned, followed by a 5-min telephone call one week later to communicate the CEA result. As 4 annual visits are planned in the first three years of follow-up and 2 visits are scheduled in the last two years of follow-up, the total amount of time per patient amounts to 5.3 h, i.e., 1067 h for a cohort of 200 patients.

In the study group, outpatient visits were reduced to once annually. This visit was planned to last 15 min. No telephone call was necessary, because the CEA communications are made automatically in writing. Work with the software consumes about 30 min a week for a cohort of approximately 200 patients. For the five years of follow-up, this means 250 h on visits plus 130 h on the software, resulting in 380 h of work. Thus, the time saved by the implementation of our software tool amounts to more than 100 working hours annually.

### Outcomes of follow-up

In the study group, 16/184 patients (9%) developed recurrent disease, occurring in the course of the 1.5 years of follow-up. The study group had a median follow-up time of 2.66 years (range 0.3 - 10.8). Curative treatment of the recurrent disease was possible in seven patients out of these sixteen (44%).

In the control group, 8/61 patients (13%) had recurrent disease in the course of the intensified follow-up scheme; the median follow-up was 3.21 years (range 0.1 - 6.1 years). Curative treatment of these metastases/local recurrences was possible in 1 out of these 8 patients (12.5%). There was no statistical significant difference in the curability of recurrent disease between groups.

## Discussion

It is known that the implementation of a new guideline among surgical specialists in a large hospital can be difficult, and the recent literature shows that software support increases adherence of the implementation [6,7]. Hereby, clinical decision support-systems using software appeared to be significantly more effective in improving clinical practice than systems that relied on manual processes [8]. In literature, we did not find any large studies on the use of software to ameliorate follow-up of colorectal cancer patients.

The most important shortcoming of our new system is of logistic origin. The software was not constructed to detect patients' outpatient visits in other medical fields. These visits can interfere with the follow-up, and were noticed later because of the reduction in outpatient visits. We are now working on this by asking patients to let us know if they have been on other outpatient or inpatient visits. This has led to a comprehensive approach to managing the data information and patient flows in our healthcare environment.

We recognize that our evaluation using structured interviews with doctors is not evidence-based but based on our own face validity; all authors have read and checked the self-constructed interview. Furthermore we realize that the construction of the groups was not randomized, which can lead to a selection bias. In our view, the lack of a national evidence-based follow-up scheme justifies the applied differences between frequencies in outpatient visits between groups. Hereby a comparison between the groups does not show differences in patient's characteristics nor in tumour stage, which strengthens our idea that our conclusions are not only valuable but also statistically solid. The survival per group did not differ significantly in the first year after implementation; of course, 3- and 5-year survival curves will be calculated by the time to evaluate the long-term outcomes.

The implementation of this software in other hospitals will be encouraged by the authors. We realize that the links between the hospital information system and the software have to be rewritten in the case of other hospital information systems, but are sure that these are minor limitations.

## Conclusions

We evaluated the experiences with and effectiveness of follow-up care for patients with colorectal cancer in a non-randomized study in a consecutive group of patients using software support, and compared this with follow-up care without the software. There was a large decrease in time spent on follow-up, while the safety in terms of finding curable recurrences was comparable.

Patients' consent to participate in the software group was high. The quick provision of information about the latest CEA value and the possibility of going to a nearby laboratory were appreciated. We did not perform a patients' satisfaction study, but a study on patients' satisfaction was performed by members of our group (IG, GB) in a pilot trial on frequent CEA testing in another Dutch hospital. The findings of psychological analysis of patients in this pilot (n = 64) show a significantly higher level of satisfaction with the outpatient follow-up scheme compared with the care usually given (response rate 84%). No significant differences were found between the trial group and the comparison group in attitude towards follow-up, anxiety, depression, and cancer worries.[Reijnen I., I. Grossmann, P. A. M. Kommers, C. H. C. Drossaert, J. M. Klaase, T. Wiggers, G. H. de Bock: Positive psychological evaluation of an intensive follow-up trial in colorectal cancer based upon high frequency serum CEA measurements, Submitted]

The program provides a graphical outline of the increase in CEA, visible for the doctors working with the software. This supports the making of clinical decisions for action in follow-up care. Strong features of our software are the absence of a need for additional data entry since the software can extract all relevant information from the hospital information system, and the integration with the hospital charting system, which can put generated patients' letters back in the system after the letters being approved, hereby reducing the clinician's workload. The use and development of a so-called decision-support tool is generally a response to poor compliance of clinicians with guidelines [9]; in our hospital it was constructed to simplify working with large patient groups receiving follow-up. Computer-based support for decision making is an accepted independent factor in improving clinical practice. We found that the software was easy to use and showed few technical problems. In interviews with clinicians, we found that the software tool was well accepted. We recognize that the lack of a validated interview with surgeons for this purpose is a weakness of our study design, but constructed the interview carefully with face validity.

The main aim of follow-up is safety in finding metastatic or local recurrences as soon as possible. We have found a similar rate of curative options for both groups, what supports the idea that outpatient visits can safely be diminished if CEA is closely monitored. We recognize that in this non-randomized study we did not have the ideal design to enable us to draw conclusions about this issue. The small number of recurrences may be a result of the improved preoperative staging over years in combination with the relatively short period of 1.5 years in which we evaluated the intensified follow-up scheme. The safety of our computer-support program has been proved; further work is now being done and is focused on finding an accurate and evidence-based follow-up system, which can then be safely and conveniently supported by the software constructed (Netherlands Trial Register: NTR 2182).

## Availability and Requirements

All data on availability and Requirements are given in Additional file 1.

## Abbreviations

CRC: Colorectal Cancer; CEA: Carcino-Embryonis Antigen; CT: Computed Tomography; NTR: Netherlands Trial Register; PHP: Hypertext Preprocessor; SQL: Structured Query Language; CSS: Cascading Style Sheets; TCP/IP: Transmission Control Protocol/Internet Protocol

## Competing interests

The authors declare that they have no competing interests.

## Authors' contributions

CJ Verberne requested for data at the Comprehensive Cancer North East Netherlands, interviewed the surgeons, collected all data from medical records and constructed the database, performed statistical analysis and generally wrote the abstract and the article. CHN wrote the software CEA watch and managed all technical problems and bugs in the software, and is still working to improve the software system. He wrote and commented on the whole manuscript. GH de Bock works as an epidemiologist. She supervised constructing the database, performed and supervised all statistical analyses and gave overall feedback on the whole manuscript. IG performs a large amount of research in the field of colorectal cancer follow-up and initiated the idea for CEA-triggered follow-up. She gave feedback on the manuscript and integrity of the data. TW is professor in surgical oncology and is one of the supervisors of this manuscript He initiated the idea of this article, and gave feedback on text and statistics. KH introduced the software into system in medical practice, worked daily with the software and gave feedback on the text and structure of the whole manuscript. All authors read and approved the final manuscript

## Consent

As this article is about a software program rather than a patient, no informed consent was necessary.

## Endnotes

The software evaluated in this study was mentioned and presented at the European School of Oncology class in Cascais, Portugal, February 2011.

## Pre-publication history

The pre-publication history for this paper can be accessed here:

http://www.biomedcentral.com/1472-6947/12/14/prepub

## Supplementary Material

Additional file 1 **CEA Watch Manual**. Click here for file
