# Supplementary material for: Evaluation of the use of decision-support software in carcino-embryonic antigen (CEA)-based follow-up of patients with colorectal cancer
Source: BMC Med Inform Decis Mak. 2012 Mar 5;12:14. doi: 10.1186/1472-6947-12-14 (PMC3330015; doi:10.1186/1472-6947-12-14)
Supplement: Additional file 1 — CEA Watch Manual. [file 1472-6947-12-14-S1.DOC]

CEA Watch Manual

Hidde Nijboer

Klaas Havenga

Version 2.0, 2010

1. Introduction 3
2. Use of CEA Watch (Log in, main screen, status) 5
3. Technical issues: server 11
4. Technical issues: database 12
5. Connection with Hospital Information System “Poliplus” 13

1. Introduction

CEA Watch is a software program that supports the management of a cohort of patients in follow-up after curative resection of colorectal carcinoma (CRC)

*CEA*

An important element of this follow-up is the frequent measurement of Carcino-Embryonic Antigen (CEA). This is a sensitive tumor marker in indicating recurrent disease. Most patients have a baseline CEA value between 0.5 and 3.0 ng/mL. A significant rise in CEA is mostly caused by recurrent disease. However rise in CEA has a low specificity: it can be caused by benign diseases such as infection and by the presence of malignancies other than CRC as well.

*Follow-up schedule*

In the University Hospital Groningen, CEA is measured 4 times a year in the first 3 years after resection of colorectal cancer, and twice a year in the 4^th^ and 5^th^ year after resection. In case of a significant rise in CEA (namely two consecutive rise of 20%), further investigations in search for a recurrence will be performed by means of a Computed Tomography (CT) scan of thorax and abdomen. In case of an unclear rise (<20% rise), patients will be asked to measure their CEA value after 6 weeks instead of three months.

*Outpatient clinic visits*

Traditionally, patients were seen at the outpatient clinic every time that CEA was measured, regardless of the CEA value. Based on literature, these visits are not proven to be useful and frequent visits have disadvantages: they are time-consuming and put a strain on hospital logistics. Furthermore it can be assumed that cancer worries amongst patients will increase.

*CEA Watch*

CEA watch is an intranet-based software program that supports frequent CEA measurements. The program is based on a database. De CEA values of all eligible patients in this database are automatically checked and presented. The interpretation of the CEA value will be performed by a doctor. The outcome of this interpretation can quickly be sent to the patient by e-mail or p-mail. In case of a significant rise in CEA, patients are called by the doctor and asked to come to the clinic to perform a CT scan. The software is protected with a log-in name and password.

CEAWatch is written and developed by C. H. Nijboer, who is a computer programmer (and is now a surgical resident in Germany). The scripts are initially based on earlier work of mr Vogelzang, programmer.

Subscription to CEA Watch

Eligible patients are patients in follow-up after curative resection of colorectal cancer that are fit for metastasectomy. When a surgeon sees an eligible patient on his outpatient clinic, he/she can inform the patient about the program, give the patient information. If the patient consents to participate in the software-based follow-up, the surgeon responsible for the database will be informed. This surgeon is Klaas Havenga, who also interprets all new CEA values.

2. Use of the software

a. log-in, main screen

CEA Watch is accessible within the University Medical Centre Groningen – intranet, at all Personal Computers with internet explorer 6 or 7.

Each night, CEA Watch connects to the central server of the hospital information system of the UMCG. This information system is called poliplus. Hereby, it collects the latest CEA values of all patients in the database. If desired, this collection of data can be repeated at every moment.

To start CEA Watch, visit [http://ceawatch.umcg.nl](http://ceawatch.umcg.nl/) from a computer within the UMCG intranet.


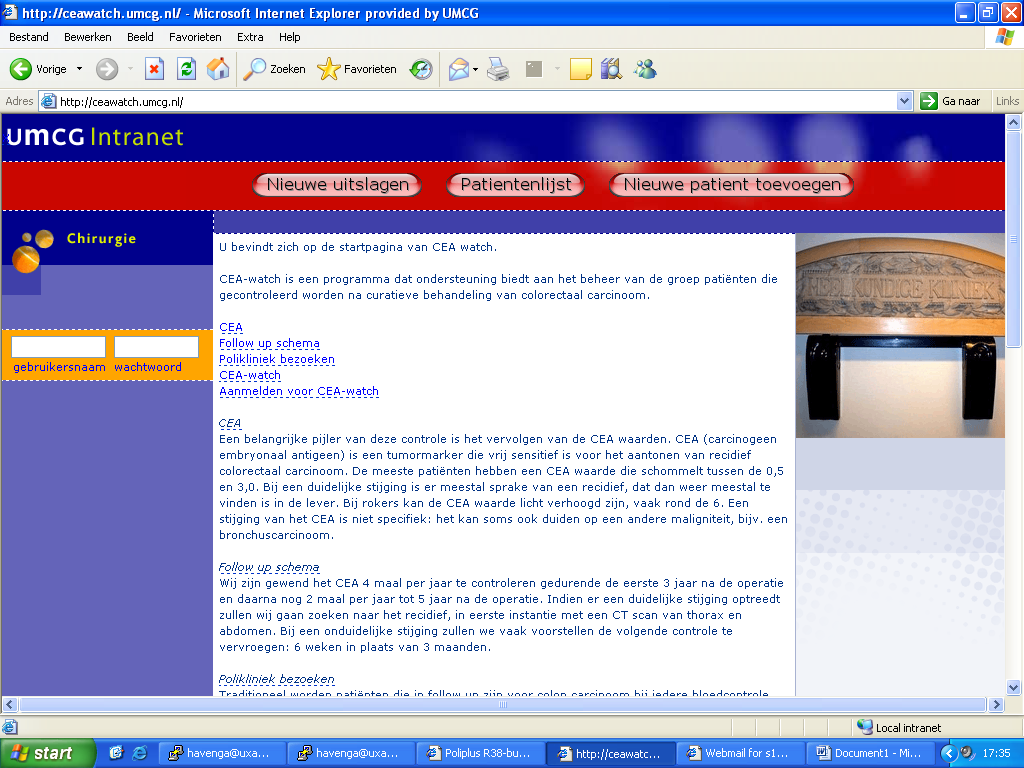


This is the first screen when entering the above-mentioned website. A log-in name and password can be provided only to workers at the UMCG. The text is in Dutch.

After logging in, this main screen will be showed; names and personal data are invisible for privacy reasons.


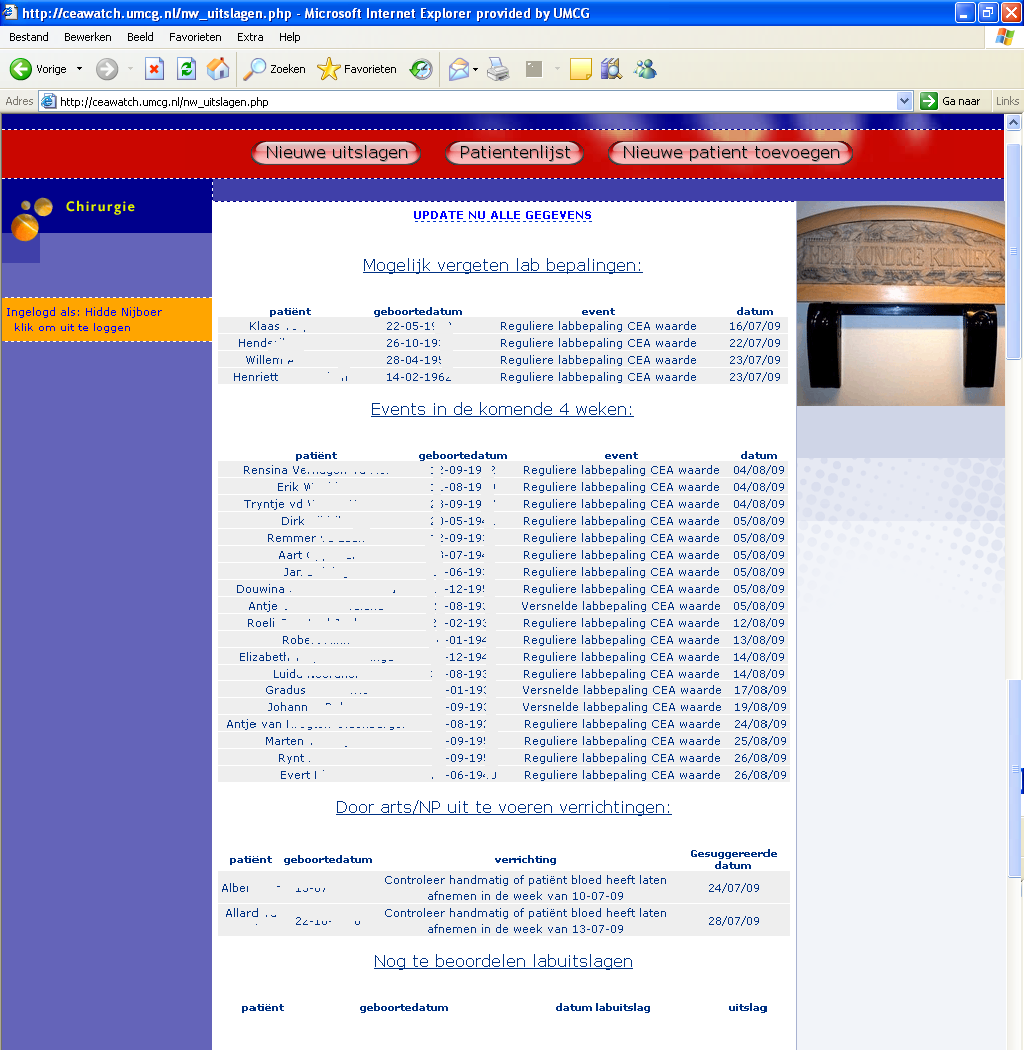


On the left, the log-in name of the user is shown; when clicking this name the user will be logged out. After 15 minutes of inactivity, the programs automatically asks for the password.

The main three buttons on top of this page indicate “New Values”, “ Patients” and “Add an new patient”. The above-shown is the “patients” screen.

When clicking “Update alle gegevens” (Dutch for update all new data) the CEA Watch server will connect to Poliplus and all new patients data (including new CEA values) will be updated. This might take a few seconds.

The button “mogelijk vergeten lab bepalingen”, which means “patients who have possibly forgotten to go to the laboratory to get their CEA checked” gives an overview of the patients who have not yet been to the laboratory within the suggested week.

The button “events in the komende 2 weken” which means “events in the upcoming 2 weeks” gives an overview of the patients for whom the next date to come to the lab to have their CEA measured lies within 2 weeks.

The button “nog te beoordelen labuitslagen” which means “CEA values that have to be checked by a doctor” gives an overview of new values to be checked on by the doctor.

**Status**


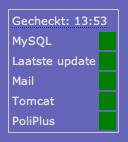


Every time that CEA Watch is used, the status will be updated, unless this update was shorter than one hour ago, or a malfunction was detected during the last update. By clicking the button “Gecheckt: tijd” which means “last ime checked” a manual check will be forced.

If all buttons are green, there are no malfunction or interruptions. The buttons turn red in case of a malfunction. In case of a malfunction of Tomcat ( which is the Java server), clicking the red button will force re-start the Java server.

- MySQL is the CEA Watch database

-Last update refers to the last overnight automatic update. A red button here can indicate maintenance of the poliplus software or hardware. By clicking the red button at Last update, a new update will be performed ( and the button will turn green)

-Mail shows the ability of CEA Watch to connect with the Mail server.

-Poliplus: if this button is red, probably there technical problems with poliplus, or planned updates or maintenance work is being performed.

b. New CEA values

In the main screen, the CEA values that still have to be interpreted are shown at the bottom. Clicking this button:


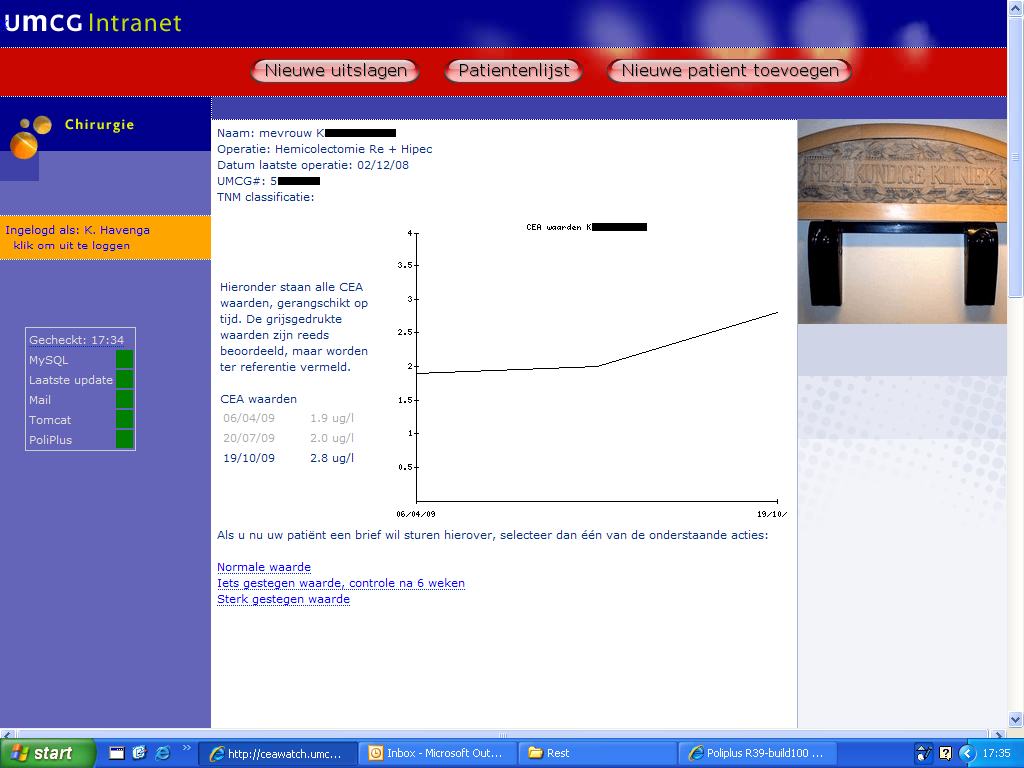


The most important data are shown here. The doctor can interpret the latest value when seeing alle the latest value and a graphical outline. Already interpreted values are shown in gray.

For interpreting the values, there are three options: the button “normale waarde”(normal value), “iets gestegen warden, controle na 6 weken” (significant increase, repeat measurement after 6 weeks), and “sterke gestegen” (significantly increased value).

Dependent on the interpretation of the value, the next step (e.g. arraging a CT scan, sending an email tot say that the value is normal) can be performed.


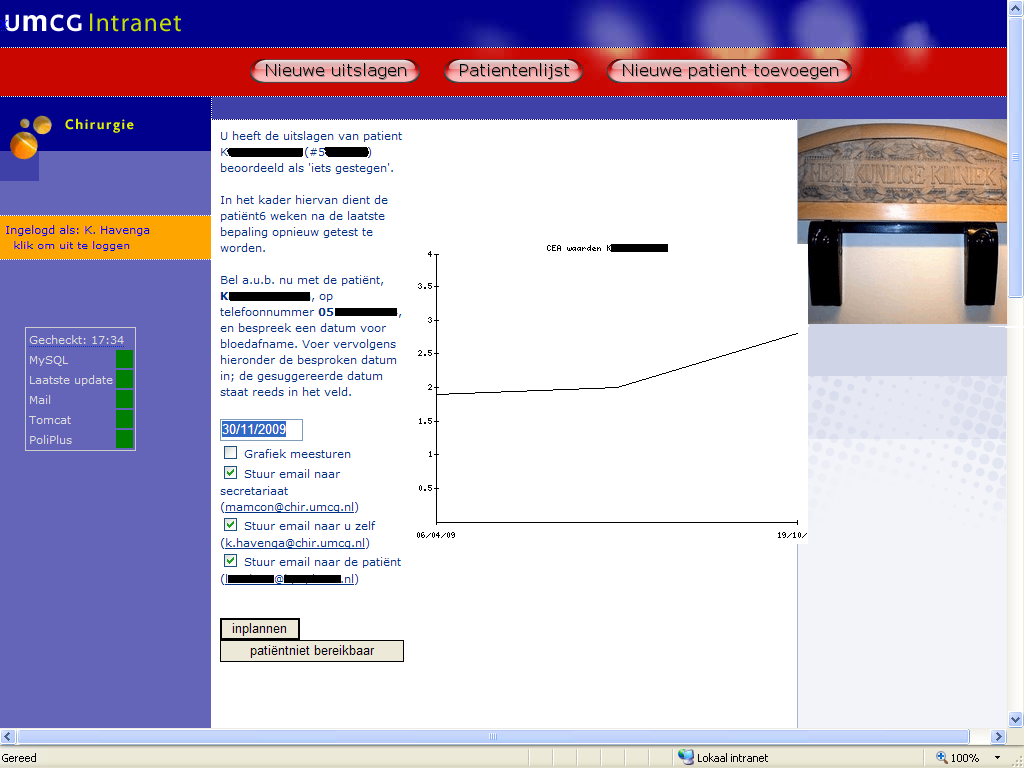


In case of a letter to the patient, the doctor can now choose where this letter is to be sent to. If the patient has consented that he/she wants to know his latest values by e-mail, this can directly be performed. The sending of a graphical outline of the latest CEA values is optional.

The next printscreen shows an example of the patients’ letter ( in Dutch)


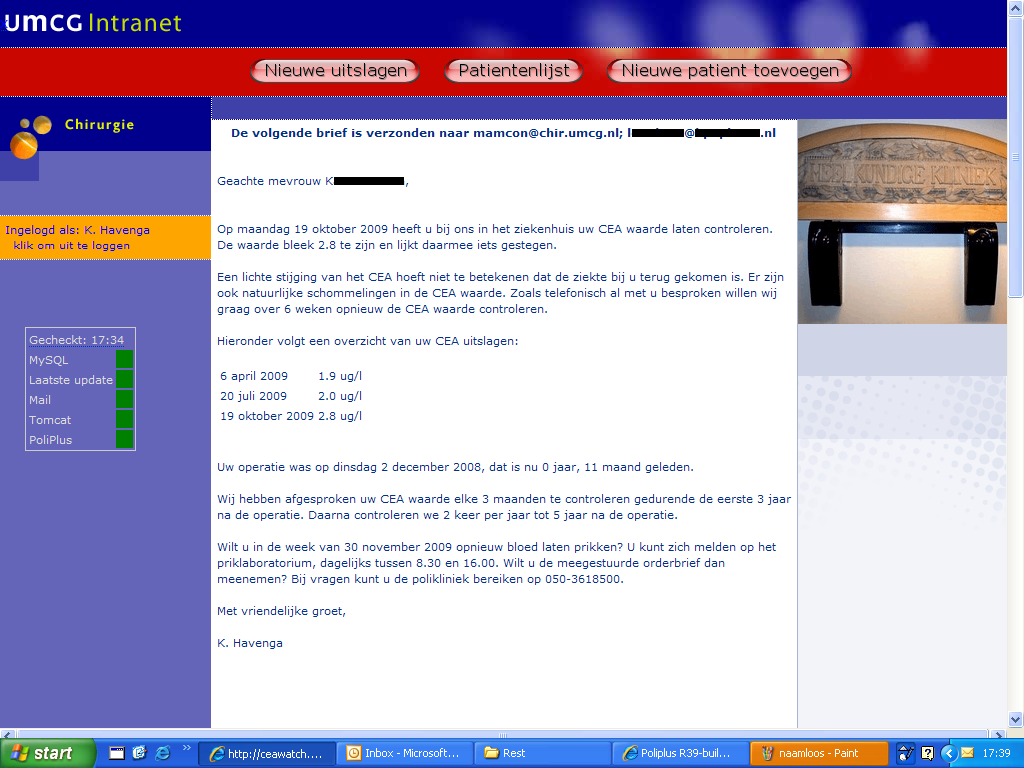


*Technical issues: server*

CEA Watch is a completely web-based program and has been tested for use in Internet Explror 6 and 7. Other browsers can not be supported ( although support is obtainable by adjusting the primary lay-out file and the CSS and Javascript-files)

CEA Watch consists of three modules:

- software package, written in PHP version 4. This looks after the primary interaction with the user. To do this, a webserver supporting PHP like Apache is needed.

- software package written in Java; this looks after the interaction between CEA watch and a hospital-information system, in this case Poliplus. It concerns servlets: programs only executing via a browser. The user does not use a direct connection with this software package: all interactions between the PHP software and the Java-servlets works with standardized HTTP connections. To do this, Apache Tomcat software is needed.

- A MySQL database, saving all data of CEA Watch. The Apache as well as the Tomcat server directly connect to this database vai TCP/IP network traffic.

Given the standardized techonology, CEA Watch can be deployed on a Windows server as well as on a linux/unix/osx server . However it is necessary that a structural Java-program can beexecuted ( for example using a program like cron), updating the database with the latest CEA values.

Needless to say, daily backup programs are essential to guarantee the well-functioning of CEA Watch.

*Database*

A MySQL-database is used to save data. Two accounts exist: the root account and the CEA Watch account, the latter one only having rights to look at the data and fill out new data.

Within the database CEA Watch several tables exist:

- myevents, in which all events are being stored. It concerns CEA values, visits to the outpatient clinic, operative procedures and so on. The fact that a CEA value has been seen and interpreted by an doctor is also been saved. What type of event a row in this table is, is been set by the colum “event_type” ( for example, “interpretation of CEA value)

All events have a unique serial number. (event_id) De colum pat_id connects the events with the patient.

-myusers: the users with their login names ( hashed by MD5)

-patient: the primary patients’table. Contains the internal pat_id, the hospital number of the patient, and personal data of the patient.

-variables: containing the various variables, for example the templates for the patient’s letters, and checking if the email system is still working. Further more the mail queue is in this table: if an email has to be sent, this will be placed in the table first and than the mailscript will be executed as a separate process ( thread). This thread extracts the patients’data out of the database. The attachments are saved in BASE64 coding.

All patients are being indentified within the system by a unique number, “pat_id”. This is not the same number as the hospital number, but a serial number.

*Connection with the hospital information system of the University Hospital Groningen (poliplus)*

The connection with poliplus exists of one “standalone” java program which takes care of the daily extraction of the new data, and a series of services that are being deployed at the apache tomcat server.

The daily update connects to the database, extracts the list of patients included in CEA Watch, and searches the latest CEA value for all patients. Hereafter, CEA values that have become known after the date of this latest CEA value are searched for within poliplus. If any, they will be added to CEA Watch. For new patients, all CEA values within the last two years are extracted and added.

The services used by CEA Watch are

-patinfo, this service is deployed at the query <http://ceawatch.umcg.nl:8080/myapps/patinfo?id=1234567>,

And as a response to this query, the patiients’ data for patient 1234567 will be extracted by the following format

<patient voorkeursnummer="1234567" patientnummer="1234567" geboortedatum="31-01-1980" geslacht="M" voornaam="Cees" voorletters="C." achternaam="Fictief" bsn="" adres="Fictiefstr 3a" postcode="9711AB" plaats="Groningen" netnummer="50" abonneenummer="3616161"/>

OKinfo (Dutch for operation info) - <http://ceawatch.umcg.nl:8080/myapps/okinfo?id=1234567>.

<operatie datum="11-9-2007" fase="postoperatief" verrichting="EERSTE CONSULT KAAKCHIRURGIE" specialismeID="MOA" reportAvailable="Nee" reportDefinitive="Nee" zittingID="0000909928" operationNr="1" operationId="0001150856" location="8"/>

<operatie datum="23-6-2005" fase="postoperatief" verrichting="BIOPSIE WEKE DELEN" specialismeID="MOA" reportAvailable="Nee" reportDefinitive="Nee" zittingID="0000690536" operationNr="1" operationId="0001567895" location="8"/>

Tomcat running: a service, answering “1” if the apache tomcat server runs without problems, and that can build a connection to poliplus.
